# Molting and cuticle deposition in the subterranean trichoniscid *Titanethes albus* (Crustacea, Isopoda)

**DOI:** 10.3897/zookeys.176.2285

**Published:** 2012-03-20

**Authors:** Miloš Vittori, Rok Kostanjšek, Nada Žnidaršič, Jasna Štrus

**Affiliations:** 1 Department of Biology, Biotechnical faculty, University of Ljubljana, Večna pot 111, SI 1000 Ljubljana, Slovenia

**Keywords:** Cuticle ultrastructure, troglobite, calcium storage

## Abstract

Terrestrial isopods are a suitable group for the study of cuticle synthesis and calcium dynamics because they molt frequently and have evolved means to store calcium during molt. Little data is currently available on molting in Synocheta and subterranean isopods. We studied the molting dynamics in the subterranean trichoniscid *Titanethes albus* under laboratory conditions and performed a microscopic investigation of sternal CaCO_3_ deposits and the tergal epithelium during molt in this species. In accordance with its lower metabolic rate, molting in the laboratory is roughly 2–3 times less frequent in *Titanethes albus* than would be expected for an epigean isopod under similar conditions. Animals assumed characteristic postures following the molt of each body half and did not consume the posterior exuviae after posterior molt. The structure of sternal calcium deposits and the ultrastructural characteristics of the epidermis during cuticle formation in *Titanethes albus* are similar to those described in representatives of Ligiidae. During the deposition of the exocuticle, the apical plasma membrane of epidermal cells forms finger-like extensions and numerous invaginations. In the ecdysial space of individuals in late premolt we observed cellular extensions surrounded by bundles of tubules.

## Introduction

Terrestrial isopods are known to molt frequently throughout their life cycle, making them particularly suitable for the study of cuticle synthesis and mineralization (Price and Holdich 1980). Furthermore, the onset of premolt can be easily determined in many terrestrial isopods due to the appearance of sternal calcium deposits (Zidar et al. 1998).

Isopods molt in two phases, first shedding the posterior and then the anterior half of the body. The boundary between the two halves is between pereionites 4 and 5. This pattern of biphasic molt is convenient and enables the simultaneous observation of the integument just prior to molt in the anterior half and just after molt in the posterior half of the same specimen.

Within Oniscidea, several studies have dealt with the ultrastructural aspects of cuticle deposition in Ligiidae (Glötzner and Ziegler 2000, Štrus and Blejec 2001) and in some members of the most terrestrial group, Crinocheta (Price and Holdich 1980, Compere 1990, Ziegler 1997). The ultrastructure of sternal CaCO_3 _deposits has been analyzed in representatives of Ligiidae and several species of Crinocheta (Ziegler and Miller 1997). Their composition and formation have been studied in great detail in *Porcellio scaber* (reviewed in Ziegler et al. 2005). Data on cuticle synthesis in Synocheta are lacking, although there is some morphological information on calcium storage in this group (Verhoeff 1926, Ziegler 2003).

Caves are stabile but nutrient poor habitats characterized by constant temperature corresponding to the average year temperature on the surface, permanent darkness and near-saturated relative humidity of air. Troglobitic animals have evolved specific adaptations to this environment, such as reduced pigmentation, thin cuticles, and lowered metabolism (Romero and Green 2005). *Titanethes albus* (C. Koch) is a large (about 1.5 cm in length) troglobitic representative of the family Trichoniscidae. The species inhabits wet limestone caves in the Dinaric Karst (Strouhal 1939) and is not exclusively terrestrial, as it is known to enter the water and can survive submerged for long periods (Sket 1986). The tergal cuticle of *Titanethes albus* is thin compared to non-troglobitic isopods of similar size. It is also less mineralized and differs from the cuticles of non-troglobitic oniscids in its mineral composition, having a lower content of magnesium and calcite (Hild et al. 2009).

In our study, we observed the temporal dynamics of molt in a laboratory culture of *Titanethes albus*. We provide an ultrastructural description of tergal cuticle deposition in this species and describe the characteristics of its sternal CaCO_3_ deposits.

## Methods

### Laboratory culture and molt cycle observations

Specimens of *Titanethes albus* from caves in central Slovenia were kept in the speleobiological laboratory at the Department of Biology, University of Ljubljana. The laboratory culture was maintained in a dark climate chamber at 11 ± 1 °C, the approximate average temperature of caves in central Slovenia. Animals were kept in glass containers with flowstone rocks, substrate from the sampling sites and spring water. Decaying wood and carrots were provided as food.

Individuals in culture were inspected for sternal deposits every month. Animals with sternal deposits were isolated into Petri dishes containing wet filter paper and observed daily. Every week, the sternal deposits were observed under a stereomicroscope and their shape was drawn. After the first molt, specimens that were not fixed for microscopic examination were kept individually separated and were inspected weekly for the presence of sternal deposits in order to determine the onset of the following premolt.

### Light microscopy and transmission electron microscopy

For ultrastructural observations, animals in premolt (determined by the presence of sternal deposits), intramolt (between the posterior and anterior molt), postmolt (1–2 days after the anterior molt), and intermolt were fixed. Individuals without sternal deposits that did not molt in the previous three weeks were considered to be in the intermolt stage.

Animals were dissected and isolated anterior tergites were fixed in a mixture of 2.5% glutaraldehyde and 2% paraformaldehyde in 0.1M cacodylate buffer (pH = 7.3) at 4 °C for at least a week. Specimens were postfixed with 1% OsO_4_ for 1 hour, dehydrated in a graded ethanol series and embedded in Spurr’s resin. Semithin (0.5 µm) sections were transferred to polylysine coated slides, stained with a mixture of Azur II and Methylene blue (Richardson et al. 1960) and imaged with an AxioImager Z.1 microscope (Zeiss) equipped with an HRc Axiocam camera. Thin (70 nm) sections were collected on copper grids, contrasted with uranyl acetate and lead citrate and examined with a CM 100 transmission electron microscope (FEI). Electron micrographs were recorded with a 792 BioScan camera (Gatan).

### Scanning electron microscopy of sternal deposits

In preparation for scanning electron microscopy of sternal deposits, sternites of pereionites 1–4 with fully developed CaCO_3_ deposits from premolt and intramolt specimens were removed, immersed in methanol and then air dried. When dry, the sternites were attached to aluminum holders and cleaved on an ultramicrotome with a glass knife. Samples were then sputter coated with platinum and imaged with a JSM-7500F field emission scanning electron microscope (JEOL).

## Results

### Duration of molt cycle

**Figure 1.**
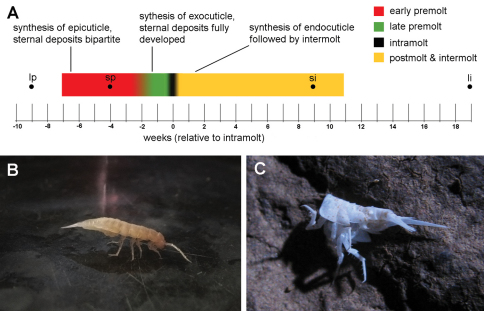
Molting in *Titanethes albus*. **A** a diagram of a typical molt cycle. The colored line shows the median observed durations of premolt and postmolt with intermolt. Different colors represent individual stages in the molt cycle. Key processes in each stage (early premolt, late premolt, and postmolt with intermolt) are indicated. Black dots indicate the onset of the longest and shortest observed premolt stages and the end of the longest and shortest observed intermolt stages **B** *Titanethes albus* immediately after posterior molt. The posterior half of the body is held upwards while the body is supported solely by the anterior four pairs of pereiopods **C** the posterior exuviae of *Titanethes albus* on a rock in Viršnica Cave. **li** end of longest observed intermolt stage **lp** onset of longest observed premolt stage **si** end of shortest observed intermolt stage **sp** onset of shortest observed premolt stage.

In the individuals studied, the median premolt duration (measured from the appearance of sternal deposits to the onset of molt) was 7 weeks (N=10). The shortest and longest premolt lasted 4 and 9 weeks, respectively. The median length of the period between the completion of molt and the second appearance of sternal deposits in non-ovigerous individuals was 11 weeks (N=9), with extreme values of 9 and 19 weeks. The only observed intermolt period of an ovigerous female lasted 34 weeks, with the release of brood after 30 weeks. The anterior ecdysis followed 3 to 5 days after the posterior ecdysis. A diagrammatic representation of a typical molt cycle is provided in Fig. 1A. Some processes indicated on the diagram will be explained later in text.

After molting the posterior exuvium, the animals hold the newly molted body-half upwards so that it does not touch the substrate (Fig. 1B). The posterior three pairs of pereiopods lie closely appressed against the ventral body surface and the entire body is supported by the anterior four pairs of pereiopods. Animals maintain this posture for several hours, but they begin using the posterior pereiopods before the onset of anterior molt. After the anterior molt, the anterior body-half is held upwards in a similar manner, with the body now supported by the posterior three pairs of pereiopods (not shown).

In the laboratory, animals never consume their posterior exuviae during intramolt and no part of the shed exoskeleton is consumed directly upon the completion of molt. Exuviae (mostly of the posterior body-half), demonstrably belonging to *Titanethes albus* due to the presence of gland-piliferous organs on the fourth pleonite, were also found on rocks in sampling localities (Fig. 1C), indicating that animals relinquish their old exoskeletons in nature as well.

### Sternal CaCO_3_ deposits

**Figure 2.**
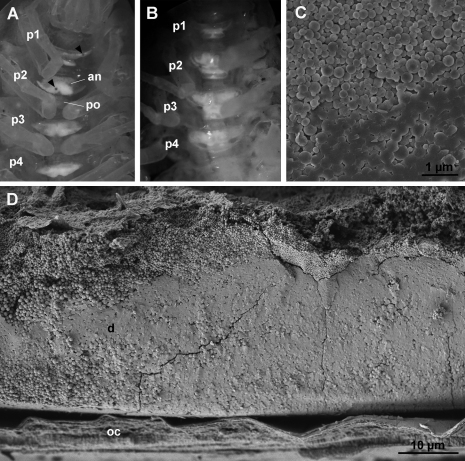
Sternal CaCO_3_ deposits in *Titanethes albus*. **A** individual in early premolt with sternal deposits on anterior four pereionites. Deposits are bipartite with a larger anterior part (**an**) and a smaller posterior part (**po**). Round fenestrations (arrowheads) perforate the deposits. **B** individual in late premolt with fully developed sternal deposits. The anterior and posterior part on each segment are fused and the deposits have a uniform shape. **C** scanning electron micrograph of spherules forming the sternal deposits in late premolt. **D** scanning electron micrograph of a cleaved sternal deposit (**d**) in late premolt. Spherules are proximally more loosely arranged. **p1** pereionite 1, **p2** pereionite 2, **p3** pereionite 3, **p4** pereionite 4, **oc** old cuticle.

Like other oniscids, *Titanethes albus* develops sternalCaCO_3_ deposits in the ecdysial space of the anterior four sternites in premolt. Initially, the deposits are bipartite, with an anterior and a posterior part on each of the first four sternites of the pereion. The shape of sternal deposits in early premolt varies greatly between individuals. The anterior part of individual deposits is always larger and symmetrical (Fig. 2A), whereas the posterior part is smaller and irregular in shape. Sternal deposits in early premolt often display small round fenestrations (Fig. 2A). The location of these fenestrations on the deposits is highly variable. After their initial appearance, the shape of sternal deposits remains unaltered throughout most of the premolt stage. Towards the end of this stage, their shape changes rapidly, the two parts of each deposit fuse and the deposits assume a characteristic, uniform shape (Fig. 2B) in most individuals. Animals molt within a week after this change in the shape of the sternal deposits.

As revealed by scanning electron microscopy, fully formed sternal deposits of *Titanethes albus* are composed of spherules, most of which measure 0.3 µm in diameter and vary in size between 0.1 µm and 1 µm (Fig. 2C). Deposits become progressively more compact from the sternal epidermis towards the old cuticle and spherules in the distal parts of deposits appear fused (Fig. 2D).

### Cuticle deposition

**Early premolt**

**Figure 3.**
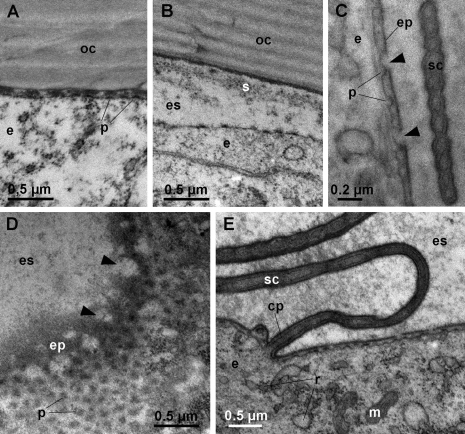
Ultrastructure of anterior tergites in early premolt. **A** apolysis. The apical surface of the epidermal cell (**e**) is detached from the old cuticle (**oc**), but the ecdysial space is narrow. The apical plasma membrane of epidermal cells forms short protrusions (**p**) with electron dense tips. **B** the ecdysial space in early premolt. A sheet of fibrous and granular material (**s**) is located in the distal part of the ecdysial space (**es**). **C** section through the apical surface of an epidermal cell (**e**) in early premolt. Gaps (arrowheads) are present in the newly formed epicuticle (**ep**) that is formed over short protrusions (**p**) of the apical plasma membrane. A developing epicuticular scale (**sc**) is visible. **D** oblique section through the apical surface of an epidermal cell in early premolt. Gaps (arrowheads) in the newly deposited epicuticle (**ep**) appear to be perforations. **E** the epidermis in early premolt. Epidermal cells (**e**) contain numerous mitochondria (**m**) and a well developed RER (**r**). Scales (**sc**) are forming around elongated projections of the apical plasma membrane (**cp**).

The onset of apolysis is observable in the anterior tergites of animals in which the sternal deposits have just appeared. The epidermis and the old cuticle are in close proximity, but protrusions of the apical plasma membrane of epidermal cells with dense tips are already evident within the narrow ecdysial space (Fig. 3A). Later, the ecdysial space is wide and a fibrous and finely granular sheet is present in its distal part (Fig. 3B). The new epicuticle is initially synthesized as a thin electron dense layer over the short protrusions of the apical plasma membrane (Fig. 3C). Gaps in the epicuticle are visible in early stages of its synthesis, indicating that it is discontinuous in the initial stages of its deposition. Oblique sections through the apical epidermal surface suggest that the gaps are perforations of the epicuticle (Fig. 3D). Epicuticular protrusions (scales or hairs) begin to form around cell projections (Fig. 3E), but are initially flat and thinner than the corresponding epicuticular structures in intermolt. Epidermal cells possess a well developed rough endoplasmic reticulum (RER) (Fig. 3E).

**Late premolt**

**Figure 4.**
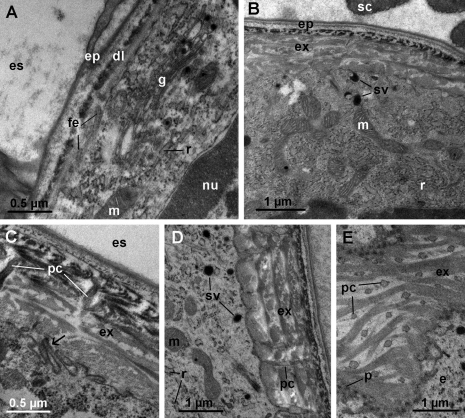
Ultrastructure of anterior tergites in late premolt and intramolt. **A** early stage of exocuticle formation. The distal dense layer (**dl**) is deposited. The apical plasma membrane of epidermal cells forms finger-like extensions (**fe**). The Golgi apparatus (**g**) is well developed. **B** anterior tergite in late premolt. Several lamellae of the new exocuticle (**ex**) are deposited. Epidermal cells contain a well developed RER (**r**), numerous mitochondria (**m**) and small electron dense vesicles (**sv**) in their apical cytoplasm. The epicuticle (**ep**) with scales (**sc**) is fully formed. **C** the apical plasma membrane of an epidermal cell in late premolt forms numerous invaginations (arrow). Cytoplasmic extensions reach into pore canals (**pc**). **D** anterior tergite in intramolt. The new exocuticle is almost fully deposited. The apical cytoplasm of epidermal cells contains electron dense vesicles (**sv**), numerous mitochondria (**m**) and a well developed RER (**r**). Long cytoplasmic extensions reach into pore canals (**pc**). **H** oblique section through the apical surface of an epidermal cell in intramolt. Pore canals (**pc**) in the new cuticle contain cytoplasmic extensions of epidermal cells. Numerous short protrusions (**p**) of the apical plasma membrane with dense tips are visible. **es** ecdysial space. **nu** nucleus.

In animals with fully formed sternal deposits, lamellae of the exocuticle are being deposited. During the synthesis of the distal dense layer (cf. Hild et al. 2009), the apical plasma membrane of epidermal cells forms finger-like extensions in addition to short protrusions (Fig. 4A). The Golgi apparatus is well developed in addition to the RER (Fig. 4A). At this stage, the epicuticle and the structures it forms are already fully developed (Fig. 4A, B).

When the exocuticle consists of 3–5 lamellae, small electron dense vesicles appear in the apical cytoplasm of epidermal cells with well developed RER and numerous mitochondria (Fig. 4B). At this stage, the apical plasma membrane of epidermal cells forms numerous invaginations in addition to protrusions (Fig. 4C). Long cytoplasmic extensions reaching into pore canals and extending to the distal dense layer of the new exocuticle become evident (Fig. 4C).

In intramolt, as the anterior tergites are nearing molt, the new exocuticle approaches its final thickness (Fig. 4D). Epidermal cells maintain the characteristics of the late premolt stage with small electron dense vesicles in the apical cytoplasm and a well developed RER (Fig. 4D). Cytoplasmic extensions in pore canals are prominent (Fig. 4D, E) and the apical plasma membrane still forms numerous short protrusions with dense tips (Fig. 4E).

**Figure 5.**
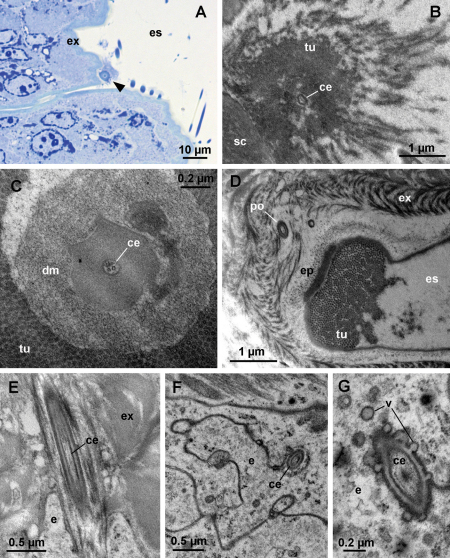
Cellular extensions and tubules in the ecdysial space. **A** oblique semithin section through the dorsal surface of an anterior tergite in intramolt. A bundle of tubules (arrowhead) on the surface of the new exocuticle (**ex**) is seen in cross-section. **B** electron micrograph of a bundle of tubules (**tu**). Proximally, the tubules are very densely arranged and they dissociate distally. A cellular extension (**ce**) within an electron dense sheath is located at the center of the bundle. **C** cross-section through a bundle of tubules. The cellular extension (**ce**) at the center of the bundle contains microtubules. Dense material (**dm**) surrounds the extension. **D** oblique section through the base of a bundle in intramolt. A pore (**po**) in the new cuticle is located beneath the bundle. Tubules (**tu**) protrude from the surface of the epicuticle (**ep**). **E** longitudinal section through a pore beneath a bundle of tubules. The pore contains a cellular extension (**ce**). **F** section though the epidermis beneath a bundle of tubules. A cellular extension (**ce**), enclosed in a sheath, is located in an invagination of an epidermal cell (**e**). **G** section through the cellular extension at the level of the epidermis. Vesicles (**v**) are present in the cytoplasm of the epidermal cell and are fused with the plasma membrane in proximity of the cellular extension. **es** ecdysial space, **sc** scale

During late premolt and intramolt stages, tubular bundles are present in the ecdysial space (Fig. 5A, B). The tubules, each measuring about 20 nm in diameter, protrude from the epicuticle in a regular arrangement and are covered by a diffuse electron dense material (Fig. 5B, C). Towards the old cuticle the regular arrangement of tubules is lost and the tubules disperse (Fig. 5B). At the center of each bundle of tubules there is a cellular extension containing parallel microtubules. The extension is enclosed in an electron dense sheath (Fig. 5C) and passes through the newly formed cuticle via a narrow pore (Fig. 5D, E). At the level of the epidermis, the cellular extension is enveloped by an epithelial cell (Fig. 5F). Small vesicles are present in the cytoplasm of the epidermal cell in the proximity of the extension. The membrane of some vesicles is continuous with the plasma membrane of the epithelial cell surrounding the extension (Fig. 5G).

**Figure 6.**
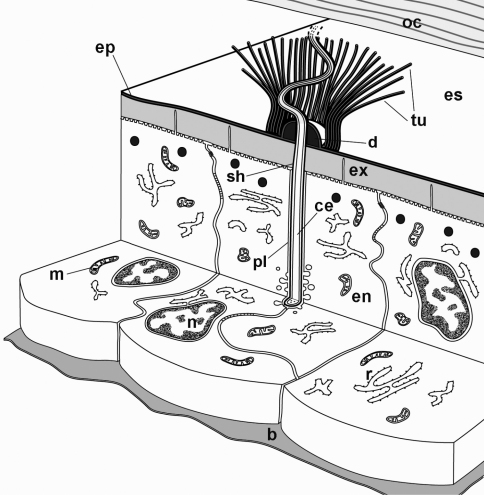
A schematic representation of cellular extensions associated with tubular bundles in the ecdysial space. A bundle of tubules (**tu**) surrounds a cellular extension (**ce**) that reaches into the ecdysial space (**es**). The cellular extension is enclosed in an electron dense sheath (**sh**). At the level of the epidermis, the cellular extension is surrounded by an enveloping epidermal cell (**en**). **b** basal lamina, **d** epicuticular thickening, **ep** new epicuticle, **ex** new exocuticle, **m** mitochondrion, **n** nucleus, **oc** old cuticle, **pl** plasma membrane of the enveloping epidermal cell, **r** RER.

A schematic representation of a tubular bundle surrounding a cellular extension and associated structures in late premolt is provided in Fig. 6.

**Postmolt**

**Figure 7.**
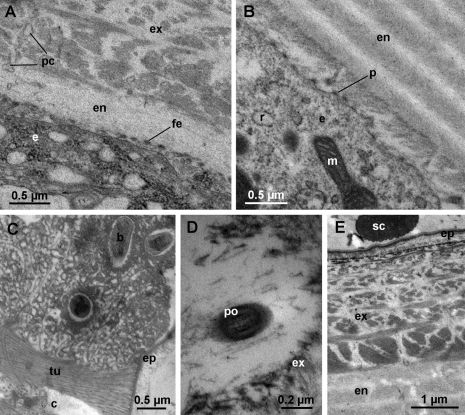
Ultrastructure of anterior tergites in postmolt. **A** anterior tergite shortly after molt. First lamellae of the endocuticle (**en**) are deposited proximally to the exocuticle (**ex**). Pore canals (**pc**) appear electron lucent. The apical plasma membrane of epidermal cells (**e**) forms finger-like extensions (**fe**). **B** apical region of an epidermal cell in postmolt. The epidermal cell (**e**) contains a well developed RER (**r**). The apical plasma membrane forms short protrusions (**p**) with dense tips. Several lamellae of the endocuticle (**en**) are deposited. **C** bundle of tubules (**tu**) protruding from the epicuticle (**ep**) of a tergite in postmolt. The center of the bundle (**c**) is electron lucent. **D** section through a pore (**po**) in the exocuticle beneath a bundle of tubules in postmolt. The lumen of the pore is electron dense. **E** The epicuticle (**ep**), exocuticle (**ex**) and endocuticle (**en**) in intermolt. **b** bacterium, **m** mitochondrion, **sc** scale.

During the first few days after anterior molt, rapid deposition of endocuticular lamellae takes place in anterior tergites (Fig. 7A, B). Small electron dense vesicles are no longer visible in the apical cytoplasm, but the RER remains well developed (Fig. 7B). The apical plasma membrane of epidermal cells still forms short protrusions with electron dense tips (Fig. 7B) and finger-like extensions may also be present (Fig. 7A). The cytoplasmic extensions in pore canals are less prominent than during intramolt and the pore canals appear electron lucent (Fig. 7A). After ecdysis, bundles of tubules remain present on the surface of the cuticle, but no cellular extensions can be observed in their proximity (Fig. 7C). The pores enabling the ensheathed cellular extensions at the centers of bundles to pass through the new cuticle in late premolt are observable in the cuticle in postmolt, but they appear very electron dense (Fig. 7D).

The fully synthesized tergal cuticle of an intermolt specimen of *Titanethes albus* is presented in Figure 7E.

## Discussion

The duration of the molt cycle as well as the length of individual stages within the cycle showed a high degree of variability in *Titanethes albus* individuals, even under laboratory conditions. A typical molt cycle in *Titanethes albus* is several times longer than in epigean isopods studied to date (Zidar et al. 1998, Štrus and Blejec 2001), but it is much shorter than that of some aquatic subterranean isopods, which molt every 9–18 months (Magniez 1975). It is difficult to compare data from studies of epigean oniscids with the results of our study, as most observations of molt cycles in terrestrial isopods were performed at room temperature while we maintained the *Titanethes albus* culture at 11°C. By observing animals at different temperatures, Steel (1980) determined the value of Q_10_ for molting frequency in *Oniscus asellus*. On the basis of his study the expected molt cycle duration in *Oniscus asellus* at 12 °C is approximately 8 weeks. The molt cycle in *Titanethes albus* under laboratory conditions is thus about two times longer than would be expected in a similarly sized epigean species at a temperature close to 11 °C. This difference corresponds to different rates of respiratory metabolism between non-troglobitic isopods and *Titanethes albus*. The expected rate of O_2_ consumption of *Oniscus asellus* at 10 °C, calculated from the previously measured metabolic rates (Phillipson and Watson 1965) and the Q_10_ for O_2_ consumption in *Oniscus asellus* (Nash 1979), would be around 0.1 ml O_2 _g^-1^h^-1^, and would therefore be roughly three times higher than the measured rate of O_2_ consumption in *Titanethes albus* at 10 °C (Simčič et al. 2010). Rates of O_2_ consumption at 10 °C for the amphibious isopod *Ligia italica* (Simčič et al. 2010) and the terrestrial species *Porcellio laevis* (Husain and Alikhan 1979) are similar to the value expected for *Oniscus asellus*. Other troglobitic crustaceans are also known to have a lower metabolic rate compared to related non-troglobitic species, which is likely to be an adaptation to the nutrient-poor cave environment (Hervant et al. 1997).

The specific postures assumed by *Titanethes albus* after the posterior and anterior molt closely resemble those described in the epigean isopod *Armadillo officinalis* (Verhoeff 1940). The lifting of the newly molted body-half from the substrate thus appears to be widespread in Oniscidea, although it has not been reported in all species studied.

Sternal deposits of *Titanethes albus*, consisting entirely of spherules, resemble the sternal deposits described in members of the family Ligiidae (Ziegler and Miller 1997) and *Titanethes albus* is the first species outside Ligiidae known to form deposits of this type. There is little data on sternal deposits in Synocheta, but it has been suggested that some representatives of the group employ three-layered deposits (Ziegler 2003). If this is the case, the absence of a proximal homogenous layer in the sternal deposits of *Titanethes albus* might represent a secondary reduction in the complexity of sternal deposits as an adaptation to the subterranean environment or to the amphibious mode of life of this species. This is further supported by the fact that three-layered deposits occur in Tylidae (Ziegler 2003), which is most likely the sister group of all other oniscids excluding Ligiidae (Schmidt 2008).

Consummation of the shed cuticle after ecdysis occurs in other crustaceans (Greenway 1985) and other arthropods, such as insects (Mira 2000). It is known that other terrestrial isopods also consume their exuviae after molting each body-half (Messner 1965, Ziegler et al. 2007). In contrast, *Titanethes albus* does not ingest the posterior exuviae. Cuticle consummation as means of obtaining calcium required for the mineralization of the anterior exoskeleton is likely less crucial for the molting *Titanethes albus*, as this species possesses very large internal calcium stores in the posterior body-half which can be utilized for cuticular mineralization after molt (personal observation). Internal calcium stores are also known to be present in some other trichoniscids (Verhoeff 1926, Ziegler 2003). It has been reported that the ligiids *Ligia hawaiensis* (Ziegler et al. 2007) and *Ligia italica* (Štrus and Blejec 2001) also do not ingest the exuviae of at least one body-half. It was shown that *Ligia hawaiensis* nevertheless retains a very high percentage of body calcium during molt which may relate to its lower body calcium content when compared to fully terrestrial isopods (Ziegler et al. 2007). Since *Titanethes albus* also has a weakly mineralized exoskeleton and lives in a moist limestone environment, it probably has a lesser need for cuticle consummation than species that must shift greater amounts of calcium to their exoskeletons over a shorter period of time without relying on environmental calcium sources.

The ultrastructural characteristics of *Titanethes albus* epidermal cells during cuticle synthesis, such as short protrusions of the apical plasma membrane, a well developed RER and abundant mitochondria throughout cuticle deposition as well as the presence of small, dense vesicles in the apical cytoplasm during exocuticle deposition are generally similar to those described in other oniscids (Price and Holdich 1980, Ziegler 1997, Štrus and Blejec 2001). Similar epithelial features are also present during molt in other crustacean groups (Koulish and Klepal 1981) and some aspects of epithelial ultrastructure during cuticle synthesis, for example the short protrusions of the apical plasma membrane, have also been found in insects (Locke 1961, 2001). In *Titanethes albus*, the apical plasma membrane of epidermal cells appears highly structured during deposition of the distal lamellae of the exocuticle. Finger-like extensions and membrane invaginations may be involved in the synthesis of the exocuticle, but they might also function in intensive transport processes between the ecdysial space and the haemolymph. In *Titanethes albus*, the epicuticle in late premolt (Fig. 3F) appears very similar to the intermolt epicuticle (Fig. 5E), indicating that there are little or no postecdysial modifications of this cuticular sublayer. This is not surprising since the epicuticular waxy layer which is modified after molt in *Oniscus asellus* (Compere 1990) is absent in the epicuticle of *Titanethes albus* (Hild et al. 2009). Also, the exocuticle in *Titanethes albus* maintains its lamellar appearance after ecdysis and the premolt exocuticle greatly resembles the intermolt exocuticle (compare Figs 3G and 5E). The exocuticle in this species is therefore not deformed by the deposition of the endocuticle, as reported in *Oniscus asellus* (Price and Holdich 1980).

The tubular structures reaching into the ecdysial space from the epicuticle in late premolt appear identical to those known from *Ligidium hypnorum* (Glötzner and Ziegler 2000) and *Ligia exotica* (Štrus et al. 2003). Tubules extending into the ecdysial space from the surface of the epicuticle therefore occur outside Ligiidae as well. In *Titanethes albus*, they are very pronounced and associate with cellular extensions reaching through the newly formed cuticle into the ecdysial space. It has been suggested that tubules within the ecdysial space of isopods may function in water retention (Glötzner and Ziegler 2000). In the case of *Titanethes albus*, their function is probably linked to cellular extensions reaching into the ecdysial space. The ensheathed, microtubule containing cellular extensions at the centers of tubular bundles resemble dendrites innervating isopod sensilla (cf. Crouau 1994), but their function remains to be established. The apparent absence of cellular extensions above the level of the new cuticle in proximity of the tubules in postmolt indicates that these are transient projections that degenerate after molt. The small vesicles associated with the cellular extensions in the epidermis suggest that intensive resorption and/or secretion of material may take place around the cell extensions at the level of the epidermis.
